# Personalized dietary management of advanced prostate cancer using nutrigenomics: a case report

**DOI:** 10.1038/s41430-023-01377-6

**Published:** 2023-12-09

**Authors:** Maree Brinkman, Hayden Green, Sam Crofts, Bridget Jones, Simone Walsh, Margaret Smith

**Affiliations:** 1 Department of Clinical Studies and Nutritional Epidemiology, Nutrition Biomed Research Institute, South Melbourne, VIC Australia; 2 Faculty of Medicine, Nursing and Health Sciences, Monash University, Clayton, VIC Australia; 3 MHTP Translation Research Facility, SmartDNA Pty Ltd, Clayton, VIC Australia

**Keywords:** Cancer epidemiology, Biomarkers

## Abstract

While there are emerging reports in the scientific literature on potential associations between cholesterol/lipids and prostate cancer, information on the dietary management of these cancer patients is currently lacking. We report on a 57-year-old white Australian male diagnosed with advanced prostate cancer who had personalized dietary management in preparation for and following his medical treatment: radiation and radical prostatectomy. Dietary recommendations were based on his blood results and nutrigenomic tests which showed a history of and genetic predisposition to dyslipidemia. Nutritional analysis also confirmed the need for dietary modification of his fat intake. Eighteen months post medical and dietary intervention his PSA level was reported at 0.1 ug/L and all blood lipid levels were within reference ranges. At two years there was no detectable disease recurrence and androgen deprivation therapy (ADT) was not required. Personalized dietary recommendations could be a clinically beneficial addition to the multidisciplinary management of prostate cancer patients.

## Introduction

Established risk factors for prostate cancer currently include: age, family history, ancestry, and genetics [1]. Modifiable risk factors include maintaining a healthy body weight, with the evidence for the role of diet in the etiology of prostate cancer still equivocal. According to the World Cancer Research Fund’s Continuous Update Project, the current level of evidence for diet is limited and suggestive only for a potentially increased risk of prostate cancer associated with a high intake of dairy and calcium foods, and low plasma levels of selenium and alpha tocopherol [1]. No other dietary factors are currently considered to have sufficient evidence to suggest either a protective or harmful influence on the development of prostate cancer, although there is emerging evidence to suggest that cholesterol which acts as a substrate for steroid hormone biosynthesis and lipid metabolism may have a role in prostate cancer cell growth, proliferation and progression [2, 3]. Dysregulation of lipid metabolism has been reported as a hallmark of prostate cancer progression with prostate cancer cells having an increased demand for de novo lipogenesis [2–4].

Here we report on a relatively young, active male who had no significant medical or family history of prostate cancer prior to diagnosis, only a history of dyslipidemia and his personalized dietary management using nutrigenomics, bloods, and dietary assessment.

## Case report

A 57-year-old white Australian male was diagnosed with advanced prostate cancer (Gleason Score: 9) in early September 2020. He had played sport at an elite level for many years and was keeping highly active at the time of diagnosis but was seeking dietary advice to help optimize the effects of his medical treatment, which included one dose of radiation (^177^Lutetium-Prostate-Specific Membrane Antigen), and surgery six weeks later involving a radical prostatectomy with pelvic lymph node dissection.

At his initial dietary consultation his self-reported weight was 88 kg, height 183 cm (estimated BMI of 26 kg/m²) and waist circumference was 84 cm. Medical history prior to diagnosis included an egg allergy as a child, intermittent sinusitis, hemorrhoids, and surgeries for sports-related injuries. Family history included obesity, diabetes, breast cancer (mother) and dyslipidemia (father).

He reported having changed his diet from the typical Western dietary pattern to a vegan diet immediately post-diagnosis. Protein intake was approximately 12% of his total energy intake and was solely from plant sources. Thus, the quantity and quality of protein intake needed to be increased to promote enhanced recovery from his upcoming radiation and surgery. Total dietary fat intake was estimated at approximately 37% of total energy intake (goal < 30%), and he was deficient in several micronutrients: vitamins B2, B12, A, zinc, and selenium.

His previous blood results were reviewed and showed a history of dyslipidemia. Nutrigenomic tests were also undertaken via the SmartDNA Genomic Wellness Test (https://www.smartdna.com.au/). He was provided with an OC-100 (DNA Genotek) Saliva Test Kit and mass array analysis was conducted on more than 167 genetic variants or SNPs, although based on his bloods, the focus was on his genetic predisposition to lipid metabolism [5].

He was provided with an initial meal plan of a high plant-based diet (approximately 75% of intake) averaging 12,000 kJ per day. Protein intake was increased to around 20% of total energy intake from both suitable animal and plant sources to ensure that he met his iron, vitamin B12 and micronutrient requirements. Other dietary recommendations focused on reducing consumption of total and saturated fat, and refined sugars.

He kept regular food diaries and dietary consultations across all phases of his active treatment and successfully underwent radiation therapy and surgery without any clinically significant issues other than some temporary general fatigue and loss of fitness. Dietary adjustment based on his food diaries helped to alleviate these symptoms e.g., slight increase in low GI carbohydrate intake.

Table 1 presents the lowered lipid levels corresponding with his dietary modifications. Results from his targeted nutrigenomic tests in Table 2 indicate a genetic predisposition to dyslipidemia, which could be managed via his personalized diet (Table 1). Total cholesterol reduced from 6.2 mmol/L to 5.1 mmol, triglycerides from 2.4 mmol to 1.0 mmol/l and LDL cholesterol from 3.8 mmol to 3.4 mmol following his dietary intervention. PSA levels are also shown to drop to 0.1ug/L following radiation and surgery. At two years post treatment there was no detectable disease recurrence or plans for ADT.

**Table 1:** Blood results for the case.

Measures (ranges)	27/06/14 Historical measure	30/06/20 Prior to diagnosis and dietary management	12/09/20 Case had started dietary modification process	20/04/21 Post radiation and surgery and after personalized dietary management program
Total Chol (3.5–5.5 mmol/L)	6.5H Total dietary fat intake estimate N/A	6.2H Total dietary fat intake estimate N/A	5.3 Total dietary fat estimate 37%	5.1 Total dietary fat estimate 28%
Tgs (<1.5 mmol/L)	2.0H	2.4H	1.0	1.0
HDL-Chol (>1.0 mmol/L)	1.25	1.29	1.15	1.28
LDL-Chol (<3.5 mmol/L)	4.3H Saturated fat intake estimate N/A	3.8H Saturated fat intake estimate N/A	3.7H Saturated fat intake estimate at 8%	3.4 Saturated fat intake estimate at 7%
Chol/HDLC (<4.5 mmol/L)	5.2H	4.8H	4.8H	4.0
Glucose (random) (3.6–7.7 mmol/L)	N/A	10.2H	N/A	N/A
Glucose (fasting) (3.6–6.0 mmol/L)	5.8	N/A	5.6	4.7
PSA (0.30–3.50 ug/L)	1.73	32H (38H at diagnosis 2 months later)	25.2H	0.1* post-surgery

*Chol* total serum cholesterol, *Tgs* Triglycerides, *HDL-Chol* high density lipoprotein cholesterol, *LDL-Chol* low density lipoprotein cholesterol, *PSA* prostate specific antigen *PSA and was the same level as of December 2021. “*H*” high and outside the reference range. *N/A* not available/not measured.

**Table 2:** Nutrigenomics test results for the case.

Metabolic Profile	Genetic Variant	Implication (Clinical Significance)	Case’s Genetic Predisposition
Lipid Metabolism	APOA1 – 5 Prime UTR Variant – rs670 – GG +/+	Associated with potential for low HDL-C. If levels are suboptimal then reducing PUFA intake to 4% of total intake has reportedly improved them. (Ordovas JM et al., AJCN, 2002).	+/+
	ABCA1 – Arg219Lys rs2230806 – GG +/+	Associated with low HDL-C levels. Exercise and maintaining a healthy body weight can improve them. (Ma XY et al., Atherosclerosis, 2011)	+/+
	LPL – Ser474Ter – rs328 - CC +/+	Associated with increased plasma VLD-C and lower HDL-C levels. Limit saturated and trans fats and avoid processed carbohydrates. (Huang X et al., Biosci Trends, 2011)	−/+
	APOB – T7673T – rs693 – AG −/+	Associated with increased risk of elevated LDL-C – need to review intake of dietary saturated fats. (Niu C et al., Lipids Health Dis, 2017)	−/+
	LDL-R – Asn591Asn – rs688 – TT +/+	Associated with elevated LDL-C levels when diet is high in saturated fat. (Jha CK et al., J Cardiovasc Dev Dis, 2018)	−/+
Metabolic Syndrome and Diabetes	TCF7L2 – Intron variant – rs7903146 – TT +/+	Associated with increased risk of developing insulin resistance and Type 2 diabetes. (Cornelis MC et al., Am J Clin Nutr 2009)	+/+
	SLC30A8 – Arg325Trp – rs13266634 – CC +/+	Associated with increased risk of developing Type 2 diabetes. (Boesgaard TW et al., Diabetologia, 2008)	−/+
Fat Absorption, Metabolism, and Obesity Risk	APOA2 – Intron variant – rs5082 – CC +/+	Associated with obesity, increased waist circumference when fat consumption is high especially when saturated fat intake is more than 10% of total calories. (Corella D et al., Arch Intern Med, 2009)	+/+
	PPARG – Pro12Ala – rs1801282 – CC +/+	Associated with increased BMI and involved with regulation of glucose and lipid metabolism and risk of Type 2 diabetes. (Mansoori A et al., Ann Nutr Metab, 2015)	+/+
Weight Management	PPARD – Intron variant – rs2016520 – AA +/+	Associated with improvement in HDL-C level with exercise. (Leonska-Duniec A et al., PLoS One, 2018)	+/+
	APOA5 – Intron variant – s662799 − TT +/+	Associated with a higher BMI and reduced weight loss when eating a diet high in fat and saturated fat. (Corella D et al., J Molecular Med 2007).	+/+

*HDL-C* high density lipoprotein cholesterol, *LDL-C* low density lipoprotein cholesterol, *VDL-C* very low-density lipoprotein cholesterol.

−/− absence of alleles associated with risk, −/+ one potential risk allele has been inherited (heterozygous) which may affect enzyme activity and biological pathway, +/+ two potential risk alleles have been inherited (homozygous) with known effects on enzyme activity and biological pathway.

## Discussion

We report on a case who received personalized dietary recommendations alongside multidisciplinary prostate cancer management, and achieved improved lipid profiles, enhanced recovery to an excellent performance status, and undetectable cancer at conclusion of our surveillance.

To date, there are only limited studies reporting on potential dietary associations with prostate cancer risk. A large prospective cohort study (Health Professionals Follow-up Study) reported that men with low cholesterol levels had a lower risk of high-grade prostate cancer/advanced disease [6]. Other studies have reported that adherence to the Western dietary pattern and high consumption of saturated fats are associated with advanced disease and higher overall and cancer specific mortality [7, 8]. There is currently even less on diet and prostate cancer management in the scientific literature, outside of maintaining a healthy body weight, eating according to the general dietary guidelines, and maintaining adequate calcium intake and vitamin D status for men on ADT [9].

To our knowledge this is the first reported case where personalized dietary management using nutrigenomics has been incorporated into the multidisciplinary management of advanced prostate cancer. Some of the limitations of this case report include investigation of a small range of targeted nutrigenomic tests which do not identify other potential risk factors for advanced prostate cancer and this being a single case’s experience which may not necessarily be generalizable. However, this case study provides important initial insights into the potentially valuable role of personalized dietary management of advanced prostate cancer. Larger studies are needed to examine the role of lipids and cholesterol in this disease and its management.

## Conclusion

Utilizing nutrigenomics, bloods and dietary data could be clinically beneficial in providing more precision-based dietary and overall management of prostate cancer patients resulting in better patient health outcomes.

## Data Availability

The original contributions presented in the study are included in the article, further inquiries can be directed to the corresponding author.
